# Platypnea-Orthodeoxia Syndrome: Two Case Reports

**DOI:** 10.7759/cureus.43807

**Published:** 2023-08-20

**Authors:** Francisca Santos, Ana Teixeira Reis, António Pessoa, Margarida Agudo, Daniela Brigas

**Affiliations:** 1 Internal Medicine, Centro Hospitalar de Setúbal, E.P.E., Setúbal, PRT

**Keywords:** upright hypoxemia, interatrial communication, right-to-left shunt, patent foramen ovale (pfo), platypnea-orthodeoxia syndrome

## Abstract

Described for the first time in the middle of the last century, platypnea-orthodeoxia syndrome (POS) is an uncommon condition of positional dyspnea and hypoxemia, triggered by standing and relieved with recumbency. It is most commonly associated with right-to-left shunting through a patent foramen ovale (PFO) or atrial septal defect, however its pathophysiology is not entirely understood. As a rare syndrome, it remains underdiagnosed in many patients. We report two different cases that illustrate the challenge of this diagnosis and therapeutic approach.

In the first case, a transesophageal echocardiogram (TEE) showed interatrial communication, ostium secundum type, with bidirectional shunting. Patient underwent a successful percutaneous closure of communication, with no residual shunting and clinical improvement and no positional hypoxemia.

In the second case, infectious complications were the cause of hemodynamic changes producing meaningful right-to-left pressure gradients, resulting in POS. After antibiotic treatment there was a major clinical improvement and a second TEE showed bidirectional shunting with no positional variation. It was assumed resolution of POS after treatment of infectious complications with no need for immediate surgery.

These two cases, with very distinctive functional and anatomic components, illustrate the challenge of understanding the exact mechanism by which POS results in clinical symptoms. A suggestive history and positional variation of oxygen saturation are very useful clues for its diagnosis in cases of unexplained hypoxemia.

## Introduction

Platypnea-orthodeoxia syndrome (POS), initially termed orthostatic cyanosis, is a rare clinical scenario identified for the first time in 1949 by Burchell et al. in a patient with post-traumatic intrathoracic arteriovenous shunt [1]. The term platypnea refers to dyspnea occurring when patient changes from decubitus to seated or upright position, whereas orthodeoxia refers to hypoxemia (and drop in oxygen saturation) precipitated by this postural change [2,3]. Together these two terms define POS, consisting of postural dyspnea and arterial oxygen desaturation that appear in standing or seated position and are solved with recumbency.

Although there is still no consensus about the magnitude of oxygen desaturation, the diagnostic criteria are fulfilled when there is a decrease in arterial oxygen pressure (PaO2) of more than 4mmHg and oxygen desaturation (SaO2) of more than 5%. In spite of this syndrome being able to occur in association with numerous conditions, there are two main causal mechanisms: a cardiogenic right-to-left (R-L) blood shunt and/or an intrapulmonary shunt and the physiopathology under POS is the mixture of deoxygenated venous blood with arterial blood, causing hypoxemia and, consequently, dyspnea [4,5].

An intracardiac shunt - an anatomical septal defect allowing communication between cardiac chambers - most commonly interatrial, is reported in more than 80% of patients with POS. Among interatrial defects, the most common is a patent foramen ovale (PFO), but communication between the two atria can also result from an atrial septal defect (ASD) or a fenestrated atrial septal aneurysm (ASA) [4-7]. Apart from an isolated intracardiac defect, most patients with POS are reported to have a concomitant cardiac, pulmonary or other extracardiac aberration that leads to right-to-left intracardiac shunt via PFO, ASD or fenestrated ASA, including aortic dilatation/aneurysm/distortion (most common), pneumectomy, diaphragm paralysis, kyphoscoliosis, abdominal surgery, pleural effusion, pulmonary fibrosis, and cardiac mass, among others [7].

Extracardiac causes of POS are mainly lung diseases that entail ventilation/perfusion mismatch or anatomic pulmonary shunt resulting from pulmonary arteriovenous malformations (AVM) [4,7]. 

This article was previously partially presented as a poster at the 18th European Congress of Internal Medicine (ECIM) on August 19-31st, 2019.

## Case presentation

Case 1

A 77-year-old man with personal history of arterial hypertension was referred to the emergency department (ED) by his cardiologist due to 84% peripheral oxygen saturation and PaO2 of 44.6 mmHg in arterial blood gases analysis. His medical management included valsartan + hydrochlorthiazide, simvastatin, pentoxifylline, and clopidogrel. Besides hypoxemia, the patient complained about a two-month dry cough and progressive dyspnoea for medium and small efforts. There was no history of tobacco use, fever, chest pain, peripheral oedema, paroxysmal nocturnal dyspnea or orthopnea. During the initial evaluation at ED, there were no chest radiographic findings like pulmonary oedema or consolidation, and, in the laboratory evaluation, there was no elevation of inflammatory parameters or D-dimers. The patient was admitted to the internal medicine ward for additional investigation of hypoxemia. 

During his stay at hospital, there were several episodes of hypoxemia needing increased oxygen supply. Additional laboratory study showed no thyroid dysfunction and no signs of an inflammatory or infectious process. N‐terminal pro‐B‐type natriuretic peptide (NT-proBNP) was within normal range. Spirometry study was normal and a thoracic angiotomography excluded pulmonary thromboembolism and other causes for respiratory failure. A ventilation-perfusion scintigraphy also excluded peripheral thromboembolism or arteriovenous malformations. Transthoracic echocardiogram documented a paradoxical movement of interventricular septum, a severe dilated left atrium and right ventricular volume overload, with no systolic dysfunction. For further surveillance, the patient was transferred to the intermediate care unit. 

**Figure 1:**
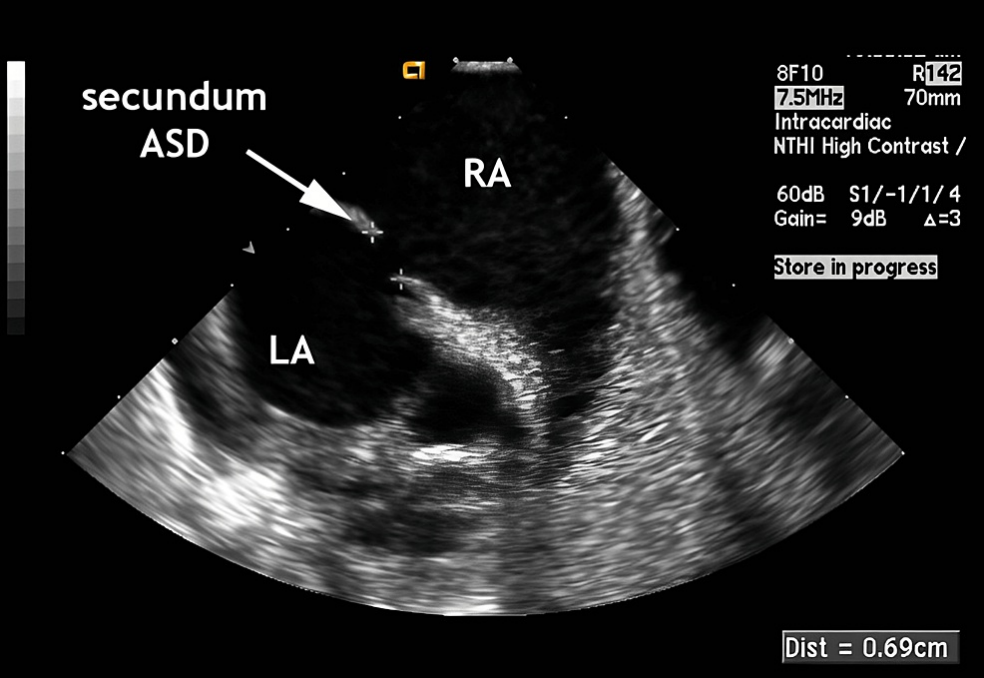
Transesophageal echocardiogram (case 1) showing interatrial communication, ostium secundum type, with 7 mm of diameter and presenting a bidirectional shunting. ASD - Atrial septal defect; LA - Left atrium; RA - Right atrium

Several episodes of severe peripheral oxygen desaturation (for values around 70 to 80%) were verified with the patient in upright position and resolved with recumbency, which was confirmed by blood gas analysis. Platypnea-orthodeoxia syndrome was suspected and a transesophageal echocardiogram (TEE) in recumbency showed, besides right ventricle volume overload, a thin and very compliant fossa ovalis* *membrane, with aneurysm criteria (possibly fenestrated) and an interatrial communication, ostium secundum type, with 7mm diameter, presenting bidirectional shunting, demonstrated by colour Doppler and agitated saline bubble-test (Figure 1). The TEE was not performed with 45º head-of-bed elevation. 

The patient underwent cardiac catheterization and an Amplatzer Septal Occluder device (Abbott, Chicago, IL, USA) was successfully implanted under intracardiac echocardiography guidance without any residual shunt. After the procedure, there was a significant clinical improvement, with no significant difference in peripheric saturation with positional variation, and the patient was discharged. Transthoracic echocardiograms three weeks and six months later continued to show a well-implanted device without any residual shunt. 

Case 2

A 53-year-old man with a 35-pack-year smoking history and obesity was admitted to the ED due to two weeks of productive cough and dyspnea that had worsened over the preceding weeks. In our emergency room, he was slightly tachycardic (heart rate 100bpm), hypertensive (arterial pressure 169/95mmHg), with polypnea and cyanosis. His room air finger oxygen saturation was 70%. Chest x-ray showed hypotransparency of both lung bases. Laboratory evaluation revealed increased reactive C protein 7mg/dL (normal level < 0.5mg/dL), no renal or thyroid dysfunction, and troponin, D-dimers and NT-proBNP levels within normal range. Arterial blood gas analysis, with fraction inspired oxygen of 31%, revealed respiratory acidaemia (pH 7,28, PaO2 68,4mmHg, PCO2 73,9mmHg). A diagnosis of pneumonia was assumed, bilevel non-invasive ventilation was started and the patient was admitted to the intermediate care unit.

Sputum culture test was negative, as well as molecular respiratory panel assay for virus detection, excluding influenza, coronavirus and other viruses. Urinary antigen tests for detection of *Streptococcus pneumonia *and *Legionella pneumophila *were also negative. 

The patient completed an antibiotic course with amoxicillin/clavulanic acid (seven days) and azithromycin (five days) with improvement of respiratory symptoms and decreasing laboratory inflammatory parameters, but there was no hypoxemia improvement. Instead, there was a need to increase ventilatory parameters and oxygen supply with no significant improvement in arterial blood gases analysis. 

A transthoracic echocardiogram showed dilated left atrium, with preserved biventricular systolic function and no diastolic dysfunction. There was no evidence of pulmonary hypertension as well. A chest CT scan excluded chronic pulmonary structural findings and a thoracic angiotomography was also performed with no evidence of pulmonary embolism or pulmonary arteriovenous malformations.

So, in the face of refractory hypoxemia, and after more frequent causes had been ruled out, platypnea-orthodeoxia syndrome was hypothesized. The patient presented with peripheral desaturation when upright (SaO2 77%) that was solved with recumbency (SaO2 95-97%). Arterial blood gases analysis corroborated this hypothesis, showing a drop of PaO2 13mmHg when patient was seated. 

**Figure 2:**
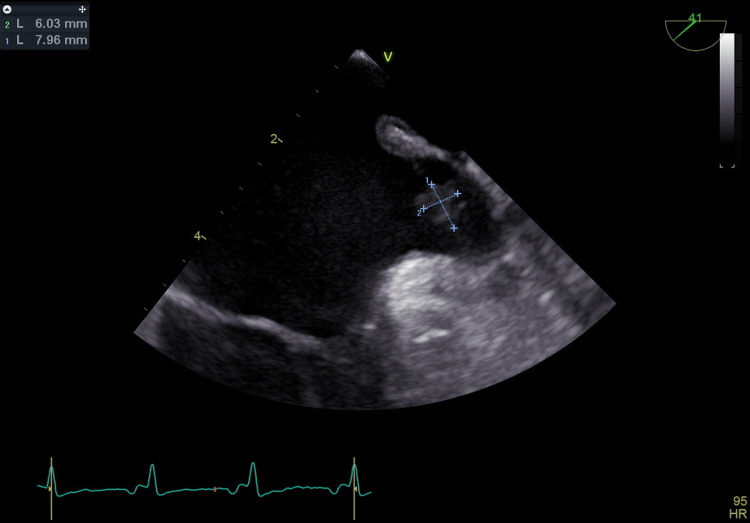
Transesophageal echocardiogram (case 2) showing left atrial appendage vegetation

In view of these findings, a TEE was performed and documented a patent foramen ovale with a right-to-left shunting accentuated with 45º head-of-bed elevation, demonstrated by color Doppler, agitated saline contrast and Valsava maneuver. The TEE also documented a left atrial appendage (LAA) vegetation (Figure 2).

Since *Staphylococcus hominis* had been isolated in sequencial blood cultures, acute endocarditis was assumed, and therapeutic dose enoxaparin and antibiotic therapy with vancomycin (30-60mg/kg/day), according to antibiogram, was promptly started. Vancomycin therapy was maintained for 34 days since blood cultures remained positive to the same agent. 

There was major clinical improvement with the referred therapeutic regime, with no significant hypoxemia, and on the 34th day, a second TEE was performed showing a free LAA and a bidirectional shunting with no positional variation. 

It was assumed resolution of POS after treatment of infectious complications and no need for immediate surgery, after discussion of the clinical case with the Heart Team committee. 

## Discussion

Although its prevalence is not established, POS is a rare clinical scenario whose diagnostic criteria include a decrease in PaO2 of more than 4mmHg and SaO2 of more than 5% [1,4]. It should be suspected in any patient presenting with dyspnea that worsens when upright and improves by assuming the lying position, especially if hypoxemia is refractory to increased oxygen support [4,7]. It can result from two main mechanisms: a cardiogenic R-L shunt - most commonly due to an interatrial communication, as PFO, ADA or ASA - and/or an intrapulmonary shunt [4-7].

In the presence of a suspicious clinical picture, the underlying etiology should be identified; even so, orthodeoxia occurs without identifiable lung or heart disease between 13% and 47% of cases, depending on different authors [6]. Since cardiac-related shunts have been the most frequently reported, an echocardiogram should be performed using contrast with agitated saline, improving the diagnostic performance. If results of transthoracic echocardiogram are inconclusive or negative, but a high index of suspicion remains, transesophageal echocardiography is recommended. At the same time intrapulmonary shunt sources can be searched with agitated saline injection. A perfusion scan scintigraphy and pulmonary arteriography should also be considered in this context [4-7].

POS secondary to intra-atrial shunting has most commonly been reported in setting of elevated right ventricular filling pressure to cause transient pressure gradient from right-to-left atrium in the upright position [4,5,7], as probably happened in our first case. 

Closure of the interatrial defect is the gold standard treatment of POS in the context of intracardiac shunting, with percutaneous closure showing symptomatic improvement in more than 95% of patients with rare adverse events and good prognosis [5]. In this situation, percutaneous approach to close the interatrial communication completely reversed the clinical picture, with no recurrence of symptoms and no residual shunt on control echocardiograms.

On the other hand, our second case seems to be a lot more complex, since the orthodeoxia occurs in the face of PFO right-to-left shunting despite of no identifiable elevation of right atrial filling pressure. 

PFO is relatively prevalent in the general population (around 25-30% depending on age). However, most people with PFO never develop symptoms of POS because the left atrial pressure is 5-8mmHg higher than the right atrial pressure resulting in functional closure with no shunting [6,7]. As PFO is relatively frequent and POS is a rare syndrome, besides the anatomical septal interruption between the two cardiac chambers, a second anatomical/ functional phenomenon is required to direct blood flow from right to left through interatrial communication [4-8].

It has been hypothesized that standing upright can cause a displacement of the interatrial septum, affecting the shape and compliance of the right atrium with interatrial communication displacement from its original position. This anatomical change re-directs venous desaturated blood from the inferior vena cava to the left atrium through the defect, whether or not a persistent Eustachian valve coexists, even in the absence of other anatomic alterations [4,7,8].

In our case the clinical improvement was mainly verified with vancomycin treatment for endocarditis atrial mass, showing a relationship between this second anatomic defect with the abnormal intracardiac through POF, despite normal right atrial pressure, as it has been described in literature [5,7]. This was corroborated by the second TEE, performed after 34 days of vancomycin therapy, showing a free LAA and a bidirectional shunting with no positional variation in opposition to what was verified in the first TEE. 

Apart from the intracardiac shunt, our second case seems to have an intrapulmonary component for POS. There are several cases reported in which POS is induced by severe ventilation/perfusion (V/Q) mismatch due to emphysema, interstitial lung diseases or consolidations, mainly if the lower pulmonary regions were predominantly affected. Due to gravity effects, this V/Q mismatch is accentuated in the upright position, leading to physiologic shunt and can present as POS. Our patient had pneumonia affecting both lung bases, which can explain the severity of the clinical picture [4,5,7].

## Conclusions

These two cases illustrate the challenge of understanding the exact mechanism by which POS results in clinical symptoms. In the first case, a percutaneous interatrial communication closure was performed, with a major long-term benefit. On the other hand, in the second case, hemodynamic factors producing meaningful right-to-left pressure gradients were predominant and symptoms were completely solved after treatment of infectious complication.

Although it is a rare entity whose diagnosis requires a high degree of suspicion, it should be suspected in patients presenting with unexplained dyspnea and arterial oxygen desaturation in orthostatism. Definitive treatment of POS due to intracardiac shunt is closure of the interatrial defect. However, there are no procedures free of risks, so this decision must be individualized according to the clinical context.
